# ANTP-SMACN7 fusion peptide alone induced high linear energy transfer irradiation radiosensitization in non-small cell lung cancer cell lines

**DOI:** 10.20892/j.issn.2095-3941.2020.0569

**Published:** 2021-09-21

**Authors:** Yi Xie, Bing Wang, Liqing Du, Yan Wang, Chang Xu, Hong Zhang, Kaixue Wen, Qiang Liu, Takanori Katsube

**Affiliations:** 1 Institute of Modern Physics, Chinese Academy of Sciences, Lanzhou 730000, China; 2 Advanced Energy Science and Technology Guangdong Laboratory, Huizhou 516029, China; 3 National Institute of Radiological Sciences, National Institutes for Quantum and Radiological Science and Technology, Chiba 263-8555, Japan; 4 Institute of Radiation Medicine, Chinese Academy of Medical Sciences & Peking Union Medical College, Tianjin 300192, China; 5 Shanxi Bethune Hospital, Shanxi Academy of Medical Science, Taiyuan 030031, China

**Keywords:** Fe-particle radiation, carbon-particle radiation, non-small cell lung cancer cells, caspase, radiosensitizer

## Abstract

**Objective:**

The aim of the present study was to investigate the mechanisms responsible for the radiation-sensitizing effect of antennapedia proteins, ANTP-SMACN7, on lung cancer cells treated with accelerated carbon and Fe particle irradiation.

**Methods:**

The ANTP-SMACN7 fusion peptide was synthesized and linked to fluorescein isothiocyanate to determine its ability to penetrate cells. A549 and NCI-H460 cells, human non-small cell lung cancer (NSCLC) cell lines, were irradiated with X-ray or high linear energy transfer (LET) irradiation with or without ANTP-SMACN7 treatment. Cellular survival, apoptosis, and protein expression were studied by colony formation assays, flow cytometry, and western blot analyses, respectively.

**Results:**

ANTP-SMACN7 fusion proteins entered the cells and promoted A549 and NCI-H460 cell high LET irradiation radiosensitization. High LET irradiation was more efficient for clonogenic cell killing and the induction of apoptosis (*P* < 0.05). Treatment with ANTP-SMACN7 significantly reduced the A549 and NCI-H460 cell clone-forming percentages and increased apoptosis through inhibition of the X-linked inhibitor of apoptosis protein and the activation of caspase-3 and caspase-9.

**Conclusions:**

Regarding pharmaceutical radiosensitization, these findings provided a way to improve high-LET clinical radiotherapy for NSCLC patients.

## Introduction

Lung cancer is the leading cause of cancer-related mortality among both males and females, with a median 5-year survival of approximately 18%. Non-small cell lung cancer (NSCLC) is the most prevalent type of lung cancer, accounting for more than 85% of cases^1,2^. The majority of patients newly diagnosed with NSCLC present with locally advanced or metastatic disease beyond the scope of a surgical cure. As a result, radiotherapy continues to be the foundation of treatment to improve survival and the quality of life for patients with lung cancer. Nevertheless, many cancer patients do not receive a satisfactory therapeutic effect because their tumors are not sensitive to ionizing irradiation, the tumor possesses prevailing malignant proliferation ability, or the patients cannot tolerate the side effects. It is therefore essential to improve the therapeutic effect of radiotherapy or reduce the required dosage without affecting the therapeutic outcome. Radiotherapy using high linear energy transfer (LET)^3^ irradiation (IR) is one promising novel strategy to control advanced cancers^4^. Carbon ion radiotherapy can achieve better 5-year local control and survival for stage I NSCLC patients than photon treatment, and is expected to be used for inoperable stage II and III NSCLC patients without severe adverse effects^3–5^.

Inhibitors of apoptosis proteins (IAPs) are essential regulators of apoptosis that prevent caspase activation or interfere with pro-apoptotic signaling intermediates, such as second mitochondrial-derived activator of caspases (SMAC)^6^. Cellular IAPs (cIAP1 and cIAP2), X-linked IAP (XIAP), ML-IAP/Livin, and survivin are frequently overexpressed in tumors, but are not expressed in most adult differentiated tissues^7^. In many malignancies, including NSCLC, gene amplification or the high expression of XIAP and survivin correlates with a poor prognosis^8–12^. Following the discovery of IAPs and their potential role in cancer cell survival, several small molecule IAP inhibitors have been developed, some of which are currently being tested in phase I and II clinical trials. Among these, SMAC mimetics have shown the greatest clinical promise to date^7,13^. However, the N-terminal 7-amino acid peptides of SMAC (SMACN7) molecules alone cannot enter the cell from the extracellular space^14^.

Studies have shown that the third alpha-helix of the homeodomain of *Drosophila* antennapedia proteins (ANTP; 16 amino acid residues from 43–58) was the minimal peptide that retained transduction functions^15^. This peptide can be used as an internalization vector for oligopeptides or oligonucleotides for transfer into the cytoplasm and nucleus of all cell types in a receptor-, channel-, energy-, and endocytosis-independent fashion^16–19^. Thus, SMACN7 can be made to enter previously impenetrable tumor cells by generating a fusion protein by attaching the C-terminal lysine of SMACN7 to an N-terminal arginine of the 16 amino acid peptide with a proline spacer. We previously synthesized a mimetic compound of SMAC, ANTP-SMACN7, in which SMACN7 was C-terminally linked to the 16 amino acid peptide from antennapedia with a proline spacer and tagged with fluorescein isothiocyanate (FITC). We further demonstrated that the ANTP-SMACN7 fusion protein could be internalized into cancer cells and could sensitize the cells to gamma rays^20^.

In the present study, using ANTP-SMACN7, we determined whether SMAC, and/or its associated mechanisms, contributed to apoptotic responses induced by high-LET IR in NSCLC cell lines. The results showed that ANTP-SMACN7 significantly promoted cell apoptosis through the inhibition of XIAP and the activation of caspase-3 and caspase-9 in lung cancer cells after irradiation with high-LET IR. These findings suggested that ANTP-SMACN7 could be a pharmaceutical candidate radiosensitizer that could function in combination with radiotherapy for the treatment of NSCLC patients.

## Materials and methods

### Cell lines

We used 2 NSCLC cell lines with significantly enhanced basal *XIAP* expressions^21^. The A549 NSCLC cell line was provided by the RIKEN BRC (Tsukuba, Japan) through the National Bio-Resource Project of the MEXT, Japan. The NCI-H460 NSCLC cell line was purchased from the American Type Culture Collection (Manassas, VA, USA). A549 and NCI-H460 cells were cultured in DMEM-High Glucose Medium (Sigma-Aldrich, St. Louis, MO, USA) and RPMI-1640 medium (Sigma-Aldrich), respectively, at 37 °C in a humidified atmosphere with 5% CO_2_. The growth media were supplemented with 10% fetal bovine serum (Gibco, Gaithersburg, MD, USA), 100 units/mL penicillin, and 3 mg/mL of streptomycin (Sigma-Aldrich). Lung cancer cell lines were tested for mycoplasma before all experiments.

### Experimental design

#### ANTP-SMACN7 fusion peptide

The ANTP (A), SMACN7 (S), and ANTP-SMACN7 (AS) recombinant peptides were synthesized by Shanghai Sangon Biotech (Shanghai, China) and contained the following amino acid sequences^20^:

ANTP, Arg-Gln-Ile-Lys-Ile-Trp-Phe-Gln-Asn-Arg-Arg-Met-Lys-Trp-Lys-Lys-FITC; SMACN7, Ala-Val-Pro-Ile-Ala-Gln-Lys-Pro-FITC; and ANTP-SMACN7, Ala-Val-Pro-Ile-Ala-Gln-Lys-Pro-Arg-Gln-Ile-Lys-Ile-Trp-Phe-Gln-Asn-Arg-Arg-Met-Lys-Trp-Lys-Lys-FITC.

#### Administration of the ANTP-SMACN7 fusion peptide

To determine whether the ANTP-SMACN7 protein could enter tumor cells, the C-terminus of the fusion peptide was labeled with FITC. According to our previous study on *in vitro* permeability, culture medium containing ANTP, SMACN7, or ANTP-SMACN7 at a concentration of 1 × 10^−5^ M was used to culture A549 and NCI-H460 cells^20^. IR of cells was performed 24 h after administration.

#### Cell irradiation

All IR procedures were performed at the National Institute of Radiological Sciences (NIRS) in Japan. The NSCLC cells were exposed to Fe (500 MeV/nucleon, original energy, LET = 200 keV/µm at the target entrance) and carbon (290 MeV/nucleon, original energy, LET = 70–80 keV/µm at the target entrance) ion beams generated by the Heavy Ion Medical Accelerator in Chiba (HIMAC) and 200 kVp X-rays (Pantak 320S; Shimadzu, Kyoto, Japan), and cellular responses to IR were then characterized. The Fe ion IR doses were 0, 0.1, 0.25, 0.5, 0.75, 1, 2, 3, and 4 Gy, whereas the carbon and X-ray IR doses were 0.45 Gy for A549 cells and 0.7 Gy for NCI-H460 cells. Each experiment was performed 3 times.

#### The colony formation assay for cell survival

T25 flasks were seeded with 2 × 10^6^ cells and cultured for 24 h; the medium was then replaced with fresh medium containing ANTP-SMACN7 and the cells were further cultured for 24 h. After IR, the cells were trypsinized and cultured in 6-well plates at a density of 400 cells per well for an additional 13–14 days (A549 cells) or 10–11 days (NCI-H460 cells) until colonies were visible and countable. The colonies were then fixed with methanol and stained with methylene blue. Colonies consisting of more than 50 cells were scored, and 3 replicate dishes were counted for each treatment^20^. The plating efficiencies were greater than 99% for A549 and NCI-H460 treatment with ANTP-SMACN7. The median lethal dose (LD_50_) was determined based on a linear-quadratic cell survival model of concentration response curves using Origin, version 8.5 (OriginLab, Northhampton, MA, USA). The survival fraction^22^ was calculated and fitted to the equation of the linear-quadratic model and expressed as follows:

$$\text{Survival}\;\text{fraction}=\exp(-\alpha \times \text{D}-\beta \times {\text{D}}^{2})$$

where D indicates the radiation dose, α is the dose effect per Gy, and β is that per Gy^2^.

#### Flow cytometric analysis of apoptosis induction

Cells were harvested at 2, 6, 12, 24, 48, and 72 h after exposure to IR and immediately fixed with 75% ethanol. For cell apoptosis analysis, the cells were stained with propidium iodide (PI, Sigma-Aldrich) staining buffer (10 mg/mL PI and 100 mg/mL RNase A) for 30 min, and fluorescence intensity was measured with a BD FACSCalibur (BD Biosciences, San Jose, CA, USA). The obtained flow cytometric data were processed using ModFit software (BD Biosciences) to determine the percentages of cellular populations in the sub-G_0_ phases.

#### Western blot analysis of the expression of apoptosis-related genes

Cell lysates were prepared with RIPA lysis buffer (50 mM Tris-HCl, 150 mmol/L NaCl, 0.1% SDS, 1% NP40, 0.5% sodium deoxycholate, 1 mmol/L phenylmethylsulfonyl fluoride, 100 µmol/L leupeptin, and 2 µg/mL aprotinin, pH 8.0). Protein extracts (20 µg) were subjected to SDS-PAGE and transferred onto polyvinylidene difluoride membranes (GE Healthcare, Chicago, IL, USA). After blocking with 5% nonfat dry milk, the membranes were incubated at 4 °C overnight with primary antibodies targeted to each of the following: XIAP (BS70748*)*, c-IAP1 (BS60459), c-IAP2 (MB0129), SMAC (BS90408), caspase-3 (BS90181), caspase-8 (BS90190), caspase-9 (BS1615), and glyceraldehyde 3-phosphate dehydrogenase (AP0063) (Bioworld, St. Louis Park, MN, USA), as well as cleaved caspase-3 (C9661), cleaved caspase-8 (C9496), and cleaved caspase-9 (20750) (Cell Signaling Technology, Danvers, MA, USA). Membranes were washed with phosphate-buffered saline (PBS) plus Tween 20 (PBST) buffer and incubated with horseradish peroxidase-conjugated secondary antibodies [Bioworld; goat anti-rabbit IgG-HRP, (BS13278) and goat anti-mouse IgG-HRP (BS12478)]. After incubation, the membranes were washed 3 times with PBST and immersed in a SuperSignal West Pico Chemiluminescent Substrate from the detection kit (Bioworld). Chemiluminescence was visualized with a ChemiDoc™ Touch Imaging system (Bio-Rad, Hercules, CA, USA) and analyzed using Image Lab™ software (Bio-Rad).

### Statistical analysis

SPSS statistical software for Windows (SPSS, Chicago, IL, USA) was used to analyze the data using either one-way analysis of variance or a Student’s *t-*test with post-hoc Tukey’s test, as appropriate; *P* < 0.05 was considered statistically significant as follows: *P  < 0.05; **P < 0.01; ***P < 0.001 All data were expressed as the mean *±* SD of the mean.

## Results

### Clonogenic survival of lung cancer cells

**Figure 1:**
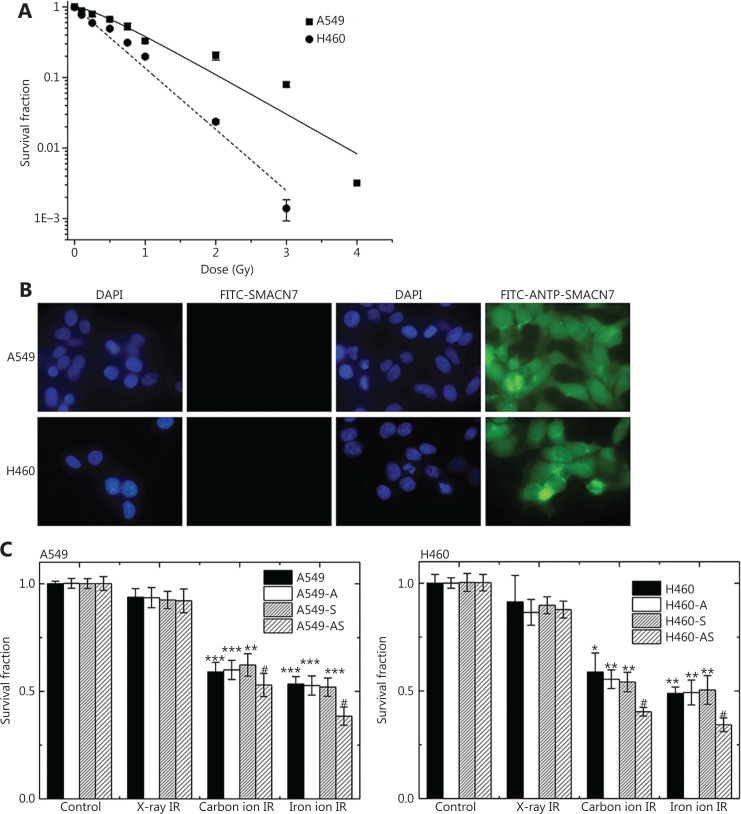
The clonogenic surviving fraction of A549 and NCI-H460 cells exposed to irradiation (IR) with or without SMACN7 addition. (A) Dose responses with respect to cell survival after exposure to high linear energy transfer (LET) IR with Fe particles. (B) Comparison of cell transduction capabilities. (C) Surviving fractions of cells treated with ANTP-SMACN7 and IR. For each type of IR, the dose delivered to A549 cells and NCI-H460 cells was 0.67 Gy and 0.43 Gy, respectively. “-A”, “-S”, and “-AS” indicate the administration of ANTP, SMACN7, and ANTP-SMACN7 peptides, respectively. “Control” represents cells without any type of IR. One-way analysis of variance followed by Tukey’s post-hoc test was used to compare quantitative parameters among X-ray, carbon ion, and Fe ion IR groups. For A549 cells, *P* < 0.001, *F* = 29.353, and for NCI-H460 cells, *P* < 0.001, *F* = 13.073. Differences between every combination of 2 groups are shown. ***P < 0.001, **P < 0.01 and *P < 0.05 indicate statistically significant differences as compared to cells with low LET X-IR without any peptides and ^#^*P* < 0.05 indicate comparisons to cells exposed to corresponding high LET IR without any peptides.

The surviving fractions of the irradiated A549 and NCI-H460 cells are shown in **Figure 1**. **Figure 1A** shows the response of A549 and NCI-H460 cells to the high LET IR with Fe particles. The LD_50_ values for A549 and NCI-H460 cells were 0.67 Gy and 0.43 Gy, respectively. We also confirmed the accumulation of FITC-tagged ANTP-SMACN7 and SMACN7 peptides in the cytoplasm and nucleus of A549 and NCI-H460 cells using fluorescence microscopy (**Figure 1B**). The control group was stained with 4′,6-diamidino-2-phenylindole solution to determine the location of the nucleus. The ANTP-SMACN7 fusion proteins successfully entered and accumulated in the cells, facilitating the pro-apoptotic effect of the fusion peptide in cells (**Figure 1B**). The results of the clonogenic responses of A549 and NCI-H460 cells to low LET IR from X-irradiation and high LET IR with carbon or Fe particles are shown in **Figure 1C**, with or without ANTP-SMACN7. For each type of IR, the doses delivered to A549 and NCI-H460 cells were 0.67 Gy and 0.43 Gy, respectively. X-irradiation at such low doses did not cause a decrease in cell survival in both cell lines, regardless of the addition of ANTP-SMACN7. In contrast, either type of high LET IR dramatically reduced cell survival in both cell lines, and administration of the ANTP-SMACN7 fusion peptide further significantly enhanced this reduction. However, SMACN7 or ANTP peptides did not have any significant effects on the clonogenic response of these cells to IR. These results indicated that at the same IR dose, high LET IR was characterized by a high relative biological effectiveness (RBE), which was higher for Fe particles than for carbon particles with respect to X-irradiation, suggesting that a safe dose of ANTP-SMACN7 could be internalized into cells to induce sensitivity to high LET IR.

### Induction of apoptosis

**Figure 2:**
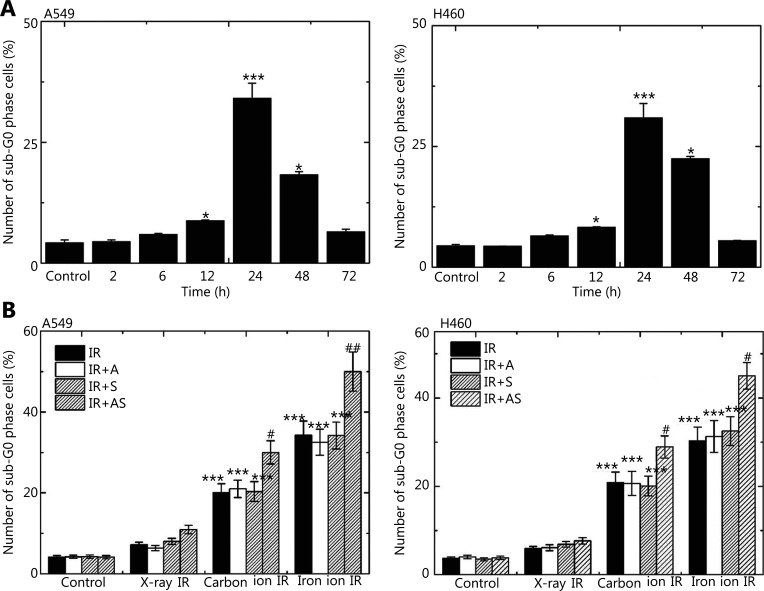
Induction of apoptosis progression by irradiation (IR) with or without SMACN7 addition. (A) Induction of apoptosis in A549 and NCI-H460 cells irradiated at doses of 0.67 and 0.43 Gy of Fe particles, respectively, at time points of 2, 6, 12, 24, 48 and 72 h after exposure, which was assessed by flow cytometry. Panel A shows the percentages of apoptotic cells (sub-G_0_ phase) at each time point. ***P < 0.001 and *P < 0.05 indicate statistically significant differences as compared to each control. (B) Percentage of apoptotic cells after exposure to low or high linear energy transfer (LET) IR in the presence of ANTP-SMACN7 at 24 h post-IR. For each type of IR, the doses delivered to A549 cells and NCI-H460 cells were 0.67 Gy and 0.43 Gy, respectively. “Control” represents cells without any type of IR. One-way analysis of variance followed by Tukey’s post-hoc test was used to compare quantitative parameters among X-ray, carbon ion, and Fe ion IR groups. For A549 cells, *P* < 0.001, *F* = 113.537, and for NCI-H460 cells, *P* < 0.001, *F* = 114.295. Differences between each combination of 2 groups are shown in (C). ***P < 0.001, as compared to cells exposed to X-irradiation without any peptides; ^##^*P* < 0.01 and ^#^*P* < 0.05, as compared to cells exposed to corresponding high LET IR without any peptides.

The time course for the induction of apoptosis mediated by high LET IR from Fe particles in the 2 cultured cell lines was analyzed by flow cytometry at 2, 6, 12, 24, 48 and 72 h after IR, based on the LD_50_ for clonogenic responses (0.67 Gy for A549 cells and 0.43 Gy for NCI-H460). PI labeling was used to detect the cellular population in the sub-G_0_ phase, indicative of apoptosis. An increase in the percentage of apoptotic cells occurred 12 h after IR and peaked at 24 h, which was significantly different compared to those in the control samples of both cell lines (**Figure 2A**). To determine whether ANTP-SMACN7 induced clonogenic cell death *via* an apoptotic mechanism, the induction of apoptosis was assessed in cells treated with ANTP-SMACN7 and IR with both low LET X-irradiation and high LET carbon or Fe particles (**Figure 2B**). For each type of IR, the doses delivered to A549 and NCI-H460 cells were 0.67 and 0.43 Gy, respectively. Similar to the results of clonogenic survival, a significant difference in apoptosis induction was not observed between the X-irradiated cells and controls (*P* > 0.05). An increase in apoptotic cells was observed after treatment with ANTP-SMACN7+X-rays as compared to the X-rays alone, but this was not statistically significant (*P* > 0.05). However, a significant increase in apoptosis induction was observed in cells irradiated with high LET IR from carbon and Fe particles 24 h after exposure. Moreover, Fe particles were more effective in inducing apoptosis than carbon particles for both cell lines (both, *P* < 0.001). In addition, concurrent treatment with ANTP-SMACN7 further promoted apoptosis induction. These results indicated that high LET IR induced a significant increase in apoptosis in lung cancer cells and that ANTP-SMACN7 further augmented this increase.

### Expression of apoptosis-related proteins

**Figure 3:**
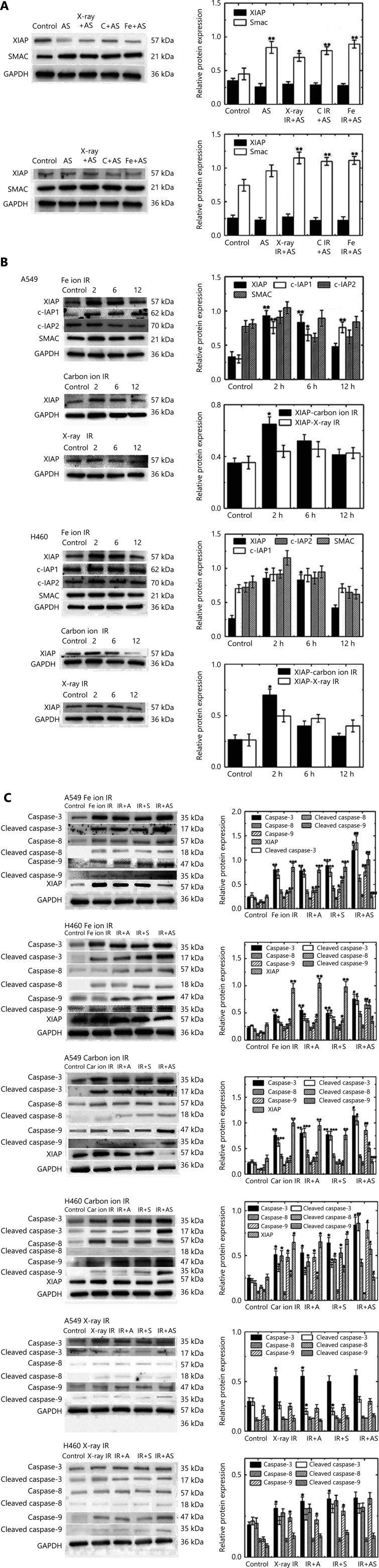
Western blot analyses of apoptosis-related proteins. (A) Expression of XIAP and SMAC in A549 and NCI-H460 cells. (B) Alterations in the expressions of XIAP, c-IAP1, c-IAP2, and SMAC in A549 and NCI-H460 cells at 2, 6, and 12 h after low and high linear energy transfer (LET) irradiation (IR). (C) Expressions and activations of caspase-3, caspase-8, and caspase-9 were examined at 2 h after exposure to low and high LET IR in the presence of ANTP-SMACN7. For each type of IR, the doses delivered to A549 cells and NCI-H460 cells were 0.67 Gy and 0.43 Gy, respectively. Bar graphs show relative band intensities of each protein standardized to that of glyceraldehyde 3-phosphate dehydrogenase, expressed in arbitrary units. One-way analysis of variance followed by Tukey’s post-hoc test was used to compare quantitative parameters among the control, IR, IR+A, IR+S, and IR+AS groups. For the A549 Fe ion IR group, *P* < 0.05, *F* = 22.619; for the A549 carbon ion IR group, *P* < 0.001, *F* = 33.828; for the A549 X-ray IR group, one-way analysis of variance was not used. For the NCI-H460 cells, *P* < 0.001, *F* = 22.031 for the Fe ion IR sub-group, *P* < 0.001, *F* = 28.083 for the carbon ion IR subgroup, and *P* < 0.001, *F* = 6.835 for the X-ray IR subgroup. Differences between each combination of 2 groups are shown in (B). ***P < 0.001; **P < 0.01; *P < 0.05 indicate statistically significant differences as compared to each control, and ^###^*P* < 0.001; ^##^*P* < 0.01; ^#^*P* < 0.05 indicate comparisons to cells exposed to IR+S.

The expressions of XIAP and SMAC were measured by Western blot 2 h after IR as shown in **Figure 3A**. SMAC expressions in the AS, AS+X-ray IR, AS+carbon ion IR, and AS+Fe ion IR groups increased significantly in A549 cells. Expressions of SMAC were also significantly upregulated in the AS+X-ray IR, AS+carbon ion IR, and AS+Fe ion IR groups of NCI-H460 cells compared to those in the controls. The expressions of XIAP, c-IAP1, c-IAP2, and SMAC proteins in A549 and NCI-H460 cells were determined by western blots at 2, 6, and 12 h after IR (**Figure 3B**). For each type of IR, the doses delivered to A549 and NCI-H460 cells were 0.67 and 0.43 Gy, respectively. The expressions of XIAP and c-IAP1 (in A549 cells) proteins significantly increased at 2 h after high LET IR and decreased with time. IR at such a low dose did not induce a significant change in the expression of XIAP at 2 h after IR. Furthermore, with low LET and high LET IR, the expression of XIAP, as well as caspase-3, caspase-8, and caspase-9, in cells was comparatively analyzed 2 h after exposure in the presence of synthetic polypeptides (**Figure 3C**). High LET IR with carbon or Fe particles was found to increase the expressions of caspase-3 and caspase-9, as well as their cleaved (activated) forms. Concurrent ANTP-SMACN7 further augmented the expressions of caspase-3 and caspase-9 and their cleaved forms with both high LET IR forms. In addition, the expression of XIAP was inhibited by ANTP-SMACN7, but not by other synthetic peptides, in both cell lines exposed to high LET IR. These results indicated that the anti-apoptotic and pro-apoptotic molecular responses were correlated with apoptotic cell killing and were consistent with the results of clonogenic cell killing. X-IR at such low doses caused only a small increase in the expression of caspase-3 and concurrently, while ANTP-SMACN7 did not have a significant effect on the expressions of these markers.

## Discussion

Evasion of apoptosis is one of the hallmarks of cancers, which contributes to the development of human tumors and therefore presents a major obstacle that makes cells resistant to anticancer treatments such as radiation therapy^23^. IAPs are anti-apoptotic proteins that are frequently overexpressed in cancer cells and associated with radioresistance^24–28^. In the current study, we investigated the effects of high LET IR and a potent radiosensitizing agent, ANTP-SMACN7, and a synthetic peptide derived from the IAP inhibitory protein, SMAC, on cell survival and apoptotic responses in 2 NSCLC lines with upregulated XIAP expressions.

Clonogenic survival analyses of these cell lines showed that A549 cells were more resistant to Fe particles than were NCI-H460 cells (**Figure 1A**). According to the results of western blot analyses by Kim et al., the upregulation of XIAP was more prominent in A549 cells than in NCI-H460 cells^21^. We obtained consistent results using similar western blot analyses (data not shown). It is likely that the enhanced expression of XIAP might correlate with the radioresistant phenotypes and the expression of IAP protein of these NSCLC cells.

We also compared the cell-killing effects of X-rays, carbon particles, and Fe particles on each cell line at fixed IR doses, when Fe particles were found to kill 50% of the cells, specifically 0.67 Gy for A549 cells and 0.43 Gy for NCI-H460 cells. Clonogenic survival assays following IR alone revealed that high LET IR killed cells more efficiently than low LET IR (**Figure 1B**). Consistently, apoptotic cells increased with LET with statistically significant differences in both cell lines (**Figure 2C**). The increase in apoptosis with LET might correlate with differences in the effectiveness of Fe and carbon particles in inducing the production of cleaved (activated) forms of caspase-3 and caspase-9^29,30^.

In addition, we also found significantly enhanced effects in terms of clonogenic cell death and apoptosis induction after combined treatment with ANTP-SMACN7 and high LET irradiation, whereas ANTP-SMACN7 alone did not exert such similar effects (**Figures 1B and 2C**). The upregulation of SMAC in the AS groups of A549 and NCI-H460 cells is shown in **Figure 3A**. SMAC disrupted the interactions between XIAP-BIR3 and caspase-9 and between Linker-BIR2 and caspase-3 or caspase-7, leading to a decrease of *XIAP*-mediated caspase inhibition. Four N-terminal AVPI residues (SMAC-4) of SMAC/DIABLO play a critical role in SMAC/DIABLO functions. Studies have shown that the AVPI sequence bound the BIR domain, which was the functional basis of the SMAC/DIABLO effects^31^. It has been shown that the 4 N-terminal residues of the caspase-9 linker peptide shared significant homology with the N-terminal tetrapeptide of mature SMAC. The inhibition of XIAP by SMAC therefore promoted caspase-9 activation and the subsequent activation of caspase-3 and apoptosis. Competition has also been noted between SMAC/DIABLO and IAP. An increase in SMAC/DIABLO concentrations therefore leads to apoptosis. However, the inhibitory effects of SMACN7 treatment at various concentrations on cancer cells occurs in a time- and concentration-dependent manner^32^. In our experiments, the level of SMAC that we chose had no apoptosis-inducing effect on cells. This concentration is thus safe for normal tissues with respect to clinical radiotherapy to treat tumor cells.

X-IR at the low doses used in this study did not cause a significant increase in the induction of clonogenic cell death and apoptosis, regardless of the addition of ANTP-SMACN7. These results indicated that high LET IR was characterized by a high RBE, which was greater for Fe particles than for carbon particles, with respect to X-irradiation. Moreover, high LET IR was found to induce a significant increase in apoptosis in NSCLC cells exhibiting XIAP overexpression. The significantly increased RBE of high LET IR might be caused by high ionization density in tracks of the heavy particles, where DNA damage becomes clustered and therefore more difficult to repair^4,33^. These results also suggested that ANTP-SMACN7 had radiosensitizing effects on cells exposed to high LET IR by contributing to the induction of apoptosis. There are 2 primary signaling cascades for apoptosis, involving the extrinsic and intrinsic pathways^27,34^. The extrinsic apoptotic pathway is triggered by binding of death ligands such as Fas ligands, TNF-α, or TRAIL to their corresponding receptors. This leads to activation of executioner caspases, involving caspase-3 and caspase-7. In contrast, intrinsic apoptotic stimuli, such as DNA damage, cause mitochondrial permeabilization and the release of apoptogenic proteins, including cytochrome *c* and SMAC, from the mitochondria into the cytosol. Cytosolic cytochrome *c* forms a large protein structure termed the apoptosome, together with apoptotic protease-activating factor 1, and activates an initiator caspase, namely caspase-9, which leads to the activation of executioner caspases, specifically caspase-3 and caspase-7. SMAC promotes apoptosis by binding to and degrading anti-apoptotic IAPs (XIAP, c-IAP1, and c-IAP2). XIAP directly binds and inhibits caspase-3, caspase-7, and caspase-9, and inhibits both intrinsic and extrinsic apoptotic pathways^35^. In contrast, c-IAP exerts its inhibitory effects on cell death indirectly *via* the ubiquitination of executioner caspases and different signaling factors involved in the extrinsic apoptotic or necrotic pathways^6,27,36^. Under most conditions, cross-talk between the extrinsic and intrinsic apoptotic pathways is minimal, and the 2 pathways usually operate independently of each other^37,38^.

In the present study, we showed, using western blot analyses, that high LET IR induced the expressions of anti-apoptotic IAPs, as well as the pro-apoptotic SMAC, caspase-3, and caspase-9 at 2 h after IR (**Figure 3**). The upregulation of XIAP might be caused by transcriptional activation, because an increase in XIAP mRNA 30 min after IR exposure has been reported in a human lymphoma cell line^39^. Our results also showed that caspase-9 was involved in extrinsic and intrinsic apoptotic pathways. The results therefore suggested that high LET IR triggered not only the intrinsic, but also the extrinsic pathways in these NSCLC cells.

**Figure 4:**
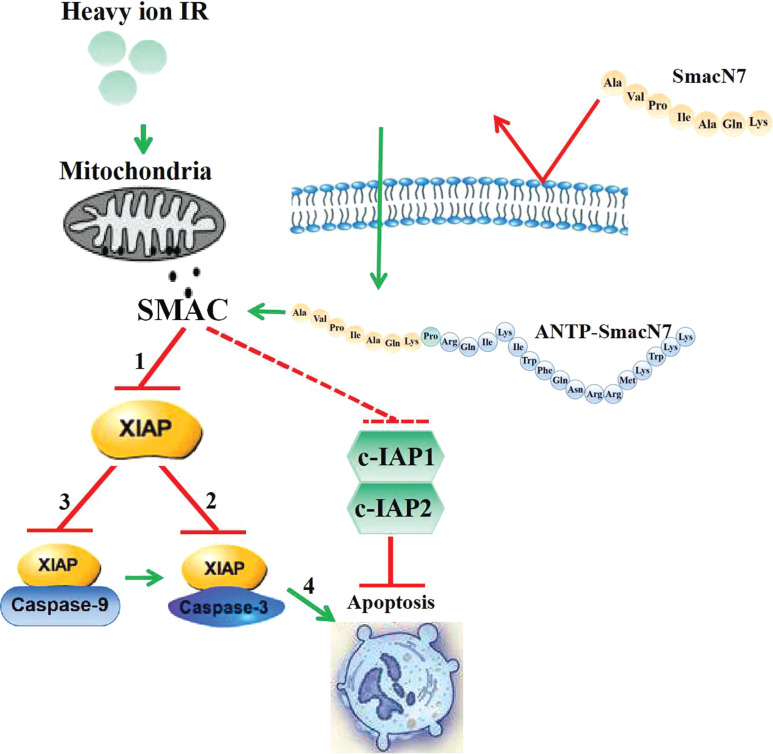
Graphic models of signal pathway illustrations in this study. ANTP-SMACN7, as a SMAC mimic, could enter into cancer cells and inhibit the activity of XIAP and then promote the activation of caspase-3 and caspase-9 in lung cancer cells after irradiation with high linear energy transfer (LET). As a result, apoptosis is increased after high LET radiation. Route 1 in this figure involves significant inhibitory effects in terms of XIAP after combined treatment with ANTP-SMACN7 and high LET IR. Inhibition of XIAP can disrupt the interactions between Linker-BIR2 and caspase-3 (route 2) and between XIAP-BIR3 and caspase-9 (route 3), leading to a relief of XIAP-mediated caspase inhibition, followed by induction increases of apoptosis (route 4). 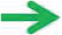 Direct action or access; 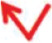 no direct access; 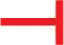 direct inhibition; 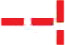 indetermination inhibition.

In the presence of ANTP-SMACN7, the expressions of caspase-3 and caspase-9 and their cleaved forms were further enhanced by high LET IR with carbon and Fe particles in both NSCLC cells, whereas the effect on caspase-8 was undetectable in A549 and NCI-H460 cells (**Figure 3B**). Furthermore, ANTP-SMACN7 abrogated the upregulation of XIAP expression induced by high LET IR (**Figure 3B**). XIAP, survivin, and BRUCE are members of the IAP family, known for their inhibitory effects on caspase activity; moreover, the dysregulation of these molecules has been widely shown to cause embryonic defects and promote tumorigenesis in humans. XIAP contains 3 BIR domains (BIR1, BIR2, and BIR3) and a single newly identified gene finger domain. As an apoptosis inhibitor, the inhibitory activities of caspase-3 and caspase-7 have been localized to the BIR2 domain, and the BIR3 domain of XIAP is responsible for inhibition of caspase-9^40,41^. In the present study, high LET IR was found to induce the upregulation of XIAP, but this change was not observed in the X-ray group because of the low doses; thus, ANTP-SMACN7 increased radiosensitivity in the presence of high LET IR. Caspase-3 and caspase-9 are direct targets of XIAP during inhibition, but caspase-8 is not^35^. In contrast, the activity of caspase-8 is indirectly modulated by c-IAP1 in the extrinsic apoptotic pathway^27,36^. **Figure 4** shows the complete signal transduction pathway of ANTP-SMACN7. Our results suggested that ANTP-SMACN7 might exert its radiosensitizing effect by inhibiting the anti-apoptotic functions of XIAP, in addition to reducing its protein levels. Accordingly, it has been reported that endogenous SMAC targeted XIAP to induce auto-ubiquitination and degradation^42^.

We also showed that ANTP-SMACN7 promoted cell apoptosis through the inhibition of XIAP and activation of caspase-3 and caspase-9 in lung cancer cells after irradiation with high-LET IR. These findings suggested that ANTP-SMACN7 might represent a possible pharmaceutical radiosensitizer for use in combination with radiotherapy for the treatment of NSCLC patients. The radiosensitization of cancer cells will ultimately lead to a reduction in the required dose for cancer radiotherapy with high LET IR, which would be beneficial in preventing side effects, including the risk of possible secondary cancers.
